# Radiation-Induced Rescue Effect: Insights from Microbeam Experiments

**DOI:** 10.3390/biology11111548

**Published:** 2022-10-23

**Authors:** Kwan Ngok Yu

**Affiliations:** Department of Physics, City University of Hong Kong, Hong Kong, China; peter.yu@cityu.edu.hk

**Keywords:** radiation biology, non-targeted effect, microbeam

## Abstract

**Simple Summary:**

The present paper introduces a radiobiological phenomenon known as the Radiation-Induced Rescue Effect (RIRE), where the radiobiological effects developed in cells irradiated with ionizing radiations are mitigated by non-irradiated cells. The primary objective of a radiotherapy treatment is to kill cancer cells with ionizing radiation while at the same time sparing the normal cells. However, RIRE was found capable of saving some of the irradiated cancer cells, so the efficacy and outcome of radiotherapy might be undermined. As such, it would be pertinent to have a better understanding of RIRE, including its underlying mechanisms and its relationships with other non-traditional radiobiological phenomena. Microbeam irradiations have some unique features that could help research on RIRE, which are explained. The paper also reviews the insights gained from previous microbeam experiments on RIRE. Some thoughts on future priorities and directions of research on RIRE exploiting unique features of microbeam radiations are presented in the last section.

**Abstract:**

The present paper reviews a non-targeted effect in radiobiology known as the Radiation-Induced Rescue Effect (RIRE) and insights gained from previous microbeam experiments on RIRE. RIRE describes the mitigation of radiobiological effects in targeted irradiated cells after they receive feedback signals from co-cultured non-irradiated bystander cells, or from the medium previously conditioning those co-cultured non-irradiated bystander cells. RIRE has established or has the potential of establishing relationships with other non-traditional new developments in the fields of radiobiology, including Radiation-Induced Bystander Effect (RIBE), Radiation-Induced Field Size Effect (RIFSE) and ultra-high dose rate (FLASH) effect, which are explained. The paper first introduces RIRE, summarizes previous findings, and surveys the mechanisms proposed for observations. Unique opportunities offered by microbeam irradiations for RIRE research and some previous microbeam studies on RIRE are then described. Some thoughts on future priorities and directions of research on RIRE exploiting unique features of microbeam radiations are presented in the last section.

## 1. Introduction

**Figure 1:**
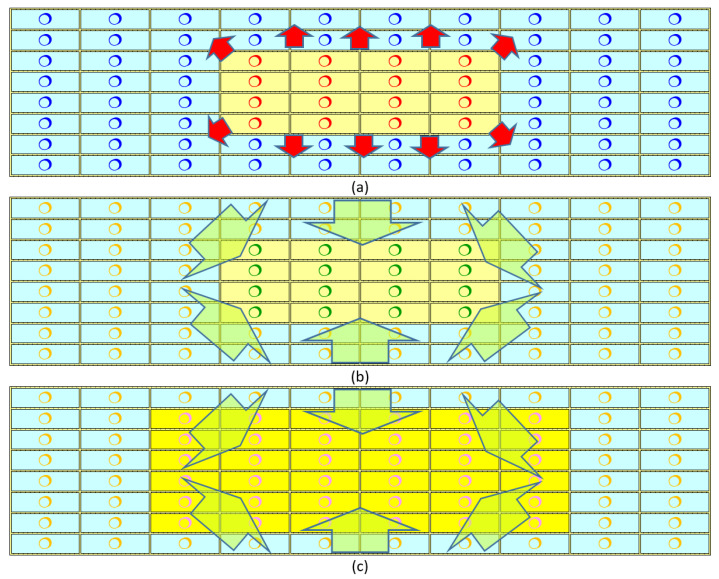
Schematic diagram to describe the characteristics of and differences among Radiation-Induced Bystander Effect (RIBE), Radiation-Induced Rescue Effect (RIRE), and Radiation-Induced Field Size Effect (RIFSE). Irradiated cells are represented by yellow rectangles; irradiated nuclei (prior to mitigation of radiobiological effects) by red circles, and rescued nuclei (subsequent to mitigation of radiobiological effects) by green and pink circles. Non-irradiated bystander cells are represented by light blue rectangles; non-irradiated nuclei prior to and subsequent to receipt of bystander signals are represented by dark blue and orange circles, respectively. Red and green arrows represent bystander and rescue signals, respectively. (**a**) Irradiated cells release bystander signals to surrounding non-irradiated bystander cells, and the ensuing effects that occur in the bystander cells are referred to as RIBE. (**b**) Non-irradiated bystander cells release rescue signals to irradiated cells, which can mitigate the radiobiological effects in the irradiated cells, and this mitigation is referred to as RIRE. (**c**) Same as (**b**) but with a different ratio between the number of irradiated and non-irradiated cells, which leads to different levels of mitigation of radiobiological effects in the irradiated cells. The dependence of radiation-induced biological effects in a cell population on the irradiation field size (in addition to the absorbed dose) is referred to as RIFSE.

Radiotherapy has been established as the most common treatment modality employed for cancer treatment. In the past decades or very recently, there have been new developments in accelerator technology that have brought to light fields of radiobiology, which might pose questions on or provide hints for improving some traditional treatment procedures. These new developments include phenomena such as the Radiation-Induced Bystander Effect (RIBE), Radiation-Induced Rescue Effect (RIRE), Radiation-Induced Field Size Effect (RIFSE), FLASH effect, etc. The present review will focus on RIRE and insights gained from previous microbeam experiments on RIRE. Interestingly, RIRE has already proven or potential relationship with all these other non-traditional phenomena, which will be discussed in the present review as well. Figure 1 provides a schematic diagram to describe the characteristics of and differences among RIBE, RIRE, and RIFSE.

The Radiation-Induced Bystander Effect (RIBE) is a phenomenon in which not only the cells directly irradiated with ionizing radiation, but also the surrounding cells that are not directly irradiated are affected by the radiation. The first report on RIBE was published by Nagasawa and Little in 1992 [1]. To date, three mechanisms underlying RIBE have been proposed, namely, (1) gap junction intercellular communication (GJIC) [2]; (2) communication of soluble signal factors among the cells through the shared medium [3]; and (3) communication of ultraviolet (UV) photons [4,5]. Interested readers are referred to published reviews on RIBE (see, e.g., refs. [6,7,8,9,10,11,12,13]).

The Radiation-Induced Rescue Effect (RIRE) was another non-targeted effect first unveiled in 2011 [14], which describes the mitigation of radiobiological effects in targeted irradiated cells after they receive feedback signals from co-cultured non-irradiated bystander cells or from the medium previously conditioning those co-cultured non-irradiated bystander cells. Previous reviews on RIRE were given by Lam et al. [15] and Yu [16]. As will be explained in Section 2 below, RIRE is intimately related to RIBE. At the time when RIRE was unveiled, its far-reaching consequences on the treatment outcome of radiotherapy was already recognized. The primary objective of a radiotherapy treatment is to kill cancer cells with ionizing radiation while at the same time sparing the normal cells. However, RIRE was found capable of saving some of the irradiated cancer cells [14]. In other words, the efficacy and outcome of radiotherapy might be affected by RIRE.

The Radiation-Induced Field Size Effect (RIFSE) refers to the phenomenon that radiobiological effects in a cell population depend on the irradiation field size and volume in addition to the absorbed dose [17]. RIFSE was reported by Coggle et al. [18] and Peel et al. [19] almost 40 years ago when they found that different β-particle doses were required for skin reactions with different irradiation field size. The relationship between RIRE and RIFSE was recently reviewed in ref. [20].

In recent years, a hot topic in the fields of radiation biology and medical physics is on the so-called FLASH effect, which refers to the lower normal tissue toxicity during radiotherapy (thereby minimizing severe acute and late effects of normal tissues) through the application of beam pulses with ultra-high dose rates (FLASH) when compared with the toxicity caused by the dose rates in traditional radiotherapy (see e.g., refs. [21,22]). This normal tissue-sparing effect was in fact described more than 40 years ago [23,24] and was rediscovered in 2014 by Favaudon et al. [21] who demonstrated normal tissue sparing and simultaneously an unchanged tumor response in mice irradiated with electrons under FLASH conditions. The effect was subsequently further confirmed for MeV electrons [25,26,27] and 100 keV X-ray photons [28]. As a result of the simultaneous lower normal tissue toxicity and unchanged tumor response, the FLASH effect allowed dose escalation and enhanced tumor control and has thus been widely touted as a promising candidate for the future in cancer radiotherapy [21,29,30].

It is now commonly believed that the FLASH effect occurs when the treatment dose is delivered within a very short period (e.g., <500 ms) and at a very high dose rate (e.g., >20 Gy s^−1^) in contrast to the dose rate in the order of Gy min^−1^ used in conventional radiotherapy [21,29]. For example, a lower normal tissue toxicity induced by electrons was achieved when doses up to 30 Gy were delivered within <500 ms [21]. In particular, the pivotal role of dose rates in the occurrence of the FLASH effect was demonstrated in previous studies. For example, Smyth et al. [31] noted the presence and absence of normal tissue sparing in mice for dose rates of ≥100 Gy s^−1^ and 37–41 Gy s^−1^, respectively. Montay-Gruel et al. [28] also revealed the non-induction and induction of memory deficits in mice or rats upon whole-brain irradiation with dose rates of ~17 kGy s^−1^ and ~0.05 Gy s^−1^, respectively.

There has been extensive interest in applying microbeam irradiation techniques to radiation biology research due to the many unique features that could be provided by such techniques. As RIRE is the focus of the present review, we will also give a survey on the insights gained from previous microbeam experiments on RIRE. Previous research studies on RIRE using microbeam irradiation systems have been performed at the National Institute of Radiological Sciences (NIRS), Chiba, Japan, and at the High Energy Accelerator Research Organization (KEK), Japan. The unique features provided by these microbeam irradiation facilities included, among others, the small beam size to target individual cells and individual cell nuclei, well-defined irradiation areas, facilitation of mixing co-cultured targeted and non-targeted cells, and delivery of ultra-high local doses and dose rates. On the other hand, however, the number of irradiated cells might be limited. Moreover, the dose rate might be exceptionally large due to the small beam size, so it might be challenging to delineate some of the radiobiological effects (e.g., to delineate RIRE from FLASH effects).

Section 2 below will first give a review on RIRE, including its discovery in 2011 [14]; RIRE for different types of ionizing radiations and cell lines/organism, and mechanisms underlying RIRE are also explained. A related phenomenon described as “bilateral bystander response” (BBR) will then be described.

Section 3 then gives a review on previous research studies on RIRE using microbeam radiations at NIRS and KEK of Japan. The unique features provided by these microbeam irradiation facilities will be reviewed at the start of the section.

Section 4 provides discussion on various issues covered in Section 2 and Section 3, or those related to using microbeam radiations to study RIRE in general, including mechanisms underlying RIRE, generalized rescue scheme, radiation dose response of RIRE, Radiation-Induced Field Size Effect (RIFSE), FLASH effect, and in vivo studies on RIRE using zebrafish embryos.

## 2. Radiation-Induced Rescue Effect (RIRE)

### 2.1. Discovery of RIRE

The first study on RIRE performed in 2011 [14] involved normal human lung fibroblasts cells and human cervical cancer (HeLa) cells and compared the responses in the targeted α-particle-irradiated cells with and without the presence of co-cultured non-irradiated bystander cells. With the presence of co-cultured non-irradiated bystander cells, the irradiated cells displayed smaller numbers of p53-binding protein 1 (53BP1) foci and micronuclei (MN), had larger surviving fractions, and fewer irradiated cells underwent apoptosis.

### 2.2. RIRE and Bilateral Bystander Response (BBR)

After the discovery of RIRE in 2011 [14], important and interesting advances were made by various research groups. In particular, in 2016, Fu et al. [32,33] reported a bilateral bystander response (BBR) where the radiobiological effects were exacerbated in the targeted human bronchial epithelial cells (Beas-2B) irradiated with α particles, after receiving feedback signals from the co-cultured non-irradiated human macrophage U937 cells (derived from histiocytic lymphoma).

It might be interesting and beneficial to differentiate this BBR [32,33] from the RIRE discovered in 2011 [14] at this early stage of the review since the two phenomena bear some resemblance in that RIRE referred to mitigation of radiobiological effects in the irradiated cells, while BBR referred to the exacerbation of radiobiological effects in the irradiated cells after the irradiated cells received feedback signals from bystander cells [16].

In association, Kong et al. [34] noted that the combination of irradiated/non-irradiated cell types in the experiments showing BBR was different from those showing RIRE. In most studies in which RIRE was detected, either (1) a single cell line was employed as both irradiated and bystander cells, or (2) cancer cells and non-transformed cells were utilized as irradiated and bystander cells, respectively, when both cell types were involved. In contrast, in the studies of Fu et al. [32,33], non-transformed (Beas-2B) cells and cancer (U937) cells were used as irradiated and bystander cells, respectively.

In order to explore whether the different combination of irradiated/non-irradiated cell types in the experiments could have led to different phenomena of RIRE and BBR, Kong et al. [34] examined the resemblance between RIRE and “metabolic cooperation” (e.g., see review in Ref. [35]), the latter referring to the cross talk between cancer cells and non-transformed cells. In certain tumor microenvironments, “metabolic cooperation” could be elicited from non-transformed cells to promote survival and growth of cancer cells [36,37,38,39,40,41,42]. Kong et al. [34] then proposed a “generalized rescue scheme” (hereafter referred to as GRS) for RIRE/BBR and metabolic cooperation, which involved “generalized stressed cells” (encompassing irradiated cells and cancer cells) and “generalized bystander cells” (encompassing those non-irradiated cells adjacent to irradiated cells and those non-transformed cells adjacent to cancer cells), in a way that they would ultimately attain the status of “adjudged stressed cells” or “adjudged bystander cells” upon partnering and that “adjudged bystander cells” would be elicited by the “adjudged stressed cells” to promote survival and growth of the latter. In other words, under this GRS, besides the “state of irradiation” of a cell type (i.e., whether it was irradiated or non-irradiated), the cell type itself (i.e., cancer cells or non-transformed cells) would also be crucial in dictating its role as the ultimate “adjudged stressed cells” (to be rescued by partnered cells) or “adjudged bystander cells” (to rescue partnered cells).

Kong et al. [34] then attempted to explain the occurrence of RIRE and BBR through the GRS between the “adjudged stressed cells” and “adjudged bystander cells”. In establishing the BBR, Fu et al. [32,33] irradiated normal human bronchial epithelial (Beas-2B) cells and found that the radiobiological effects were exacerbated, instead of being reduced, upon partnering with bystander non-irradiated human macrophage (U937) cells derived from histiocytic lymphoma. Under the GRS, the U937 cells played the role of “generalized stressed cells” according to the cell type when compared to the Beas-2B cells, while the Beas-2B cells also played the role of “generalized stressed cells” because they were irradiated and the U937 cells were non-irradiated. Apparently, any one of these two types of “generalized stressed cells” which was ultimately dictated by their rivals to promote survival and growth of the latter in the end would exhibit exacerbated effects when compared to the corresponding effects in the absence of their rivals. The GRS would then explain the exacerbated radiobiological effects in the irradiated non-transformed Beas-2B cells, which were purported to have been further dictated by the non-irradiated U937 cells to promote survival and growth of the latter.

This GRS might also help explain intriguing observations in some previous studies. For example, Widel et al. [43] detected RIRE between non-irradiated normal human dermal fibroblasts (NHDF) and the co-cultured irradiated human melanoma (Me45) cells but did not detect such RIRE between non-irradiated Me45 cells and co-cultured irradiated NHDF or co-cultured irradiated Me45 cells. Apparently, the cell type (in this case the Me45 cells in control) dominated over the state of irradiation in adjudging the ultimate “adjudged stressed cells”. Desai et al. also reported that for irradiated human lung adenocarcinoma (A549) cells, a much stronger RIRE was provided by bystander human lung normal fibroblast (WI38) cells than bystander A549 cells [44], which again suggested that the cell type dominated over the state of irradiation in adjudging the ultimate “adjudged stressed cells”.

### 2.3. RIRE for Different Types of Ionizing Radiations and Cell Lines/Organism

Following the revelation of RIRE in 2011 using α particles [14], various research groups further demonstrated RIRE through different cell lines/organism and different types of ionizing radiations. The following reports have been listed first according to whether they are broad-beam or micro-beam irradiation and then according to the type of ionizing radiation involved. Within each type of ionizing radiation, the reports will then be listed in chronological order.

Photon (X/γ-ray)-induced RIRE: Widel et al. reported that non-irradiated NHDF cells rescued partnered human melanoma (Me45) cells irradiated with 6 MV X-rays, but non-irradiated Me45 cells did not rescue partnered irradiated Me45 or NHDF cells [43]. Pereira et al. demonstrated that non-irradiated embryonic zebrafish fibroblast (ZF4) cells rescued partnered ZF4 cells irradiated with ^137^Cs γ-rays [45]. Kong et al. found that non-irradiated HeLa cells rescued partnered HeLa cells irradiated with 200 kV X-rays through a 2 mm Al filter, especially if autophagy was pre-induced in the non-irradiated HeLa cells before partnering [34]. Matsuya et al. performed half-field experiments (50% irradiated cells and 50% non-irradiated cells) and showed that non-irradiated WI-38 fibroblasts or HBEC-3KT bronchial epithelial cells rescued their counterparts irradiated with 6 MV X-rays [46]. Pathikonda et al. further confirmed that non-irradiated HeLa (cervical adenocarcinoma), MCF7 (mammary gland adenocarcinoma), CNE-2 (nasopharyngeal carcinoma), and HCT116 (colorectal carcinoma) cells rescued their partnered counterparts (same cell lines) irradiated with 200 kV X-rays through a 2 mm Al filter [47]. Adrian et al. employed 120 kV X-rays through a 2 mm Al filter to examine responses of the human prostate cancer DU-145 cell line and the human melanoma MM576 cell line and proposed that RIRE could potentially affect the results of traditional colony formation assays [48], but further confirmation might be needed [16].

[β-particle + photon (γ-ray)]-induced RIRE: Matsuya et al. revealed that non-irradiated normal human lung cells (WI-38 fibroblasts or HBEC-3KT bronchial epithelial cells) rescued their partnered irradiated counterparts within a region of the cell population irradiated non-uniformly with β-particles and γ-ray photons from a ^137^Cs- and ^134^Cs-bearing microparticle [46].

α-particle-induced RIRE: He et al. corroborated that non-irradiated hepatocyte HL-7702 cells rescued partnered human macrophage U937 cells irradiated with α particles [49]. Lam et al. verified that human cervical cancer HeLa cells or mouse embryo fibroblast NIH/3T3 cells irradiated with α particles were rescued either through co-culturing with their non-irradiated counterparts (same cell lines), or through treatment with the medium having conditioned the non-irradiated counterparts which had previously partnered with the corresponding irradiated cells [50,51].

Microbeam-proton-induced RIRE: The main objective of the present paper is to review previous research on RIRE through microbeam experiments, so these experiments and the associated results will be described in more detail in the next section and will thus only be briefly introduced here. To date, all experiments demonstrating microbeam-proton-induced RIRE have been carried out at the Single Particle Irradiation System to Cells (SPICE) at NIRS. All these experiments were carried out using microbeam protons (energy ~3.4 MeV; LET ~11.7 keV/μm; beam intensity ~1.0 × 10^4^ cps, 500 protons per cell in 0.05 s; beam diameter ~2 μm) supplied by the SPICE. Desai et al. [44] and Kobayashi et al. [52] established that non-irradiated human lung normal fibroblast (WI38) cells rescued partnered lung adenocarcinoma (A549) cells irradiated with microbeam protons. Liu et al. investigated RIRE between cancer stem-like cells (CSCs) and nonstem-like cancer cells (NSCCs) of the human fibrosarcoma HT1080 and revealed that non-irradiated CSCs rescued partnered NSCCs irradiated with microbeam protons [53]. Choi et al. reported that non-irradiated cells rescued the cells irradiated with microbeam protons in zebrafish embryos (*Danio rerio*), irradiated at the two-cell stage at 0.75 h post fertilization (hpf) [54].

Microbeam-X-ray-induced RIRE: The experiments on microbeam-X-ray-induced RIRE will be presented in fuller detail in the next section and will only be briefly described here. The studies demonstrating microbeam-X-ray-induced RIRE have been carried out at the Photon Factory of KEK [17]. These experiments were carried out using monochromatic X-rays generated by the synchrotron X-ray microbeam irradiation system installed at the BL-27B station in KEK (energy ~5.35 keV; photon fluence ~10^14^ photons m^−2^s^−1^; absorbed dose ~0.25 Gy s^−1^; beam area from 0.02 to 1.89 mm^2^). Ojima et al. established that non-irradiated primary normal human lung fibroblasts (MRC-5) rescued their partnered counterparts within irradiated fields with sizes of 0.02, 0.09, 0.81, or 1.89 mm^2^ through demonstration of the radiation-induced field size effect (RIFSE), i.e., variation of radiobiological response of cells with the irradiation field size [17].

For more convenient referencing, the most relevant information for these reports has been summarized in Table 1, which includes radiation type, doses to irradiated cells, combination of bystander/rescued cells, experimental end points, rescue responses, and authors/year/references.

### 2.4. Mechanisms Underlying RIRE

#### 2.4.1. Involvement of Cyclic Adenosine Monophosphate (cAMP)

As described in Section 2.3 above, He et al. succeeded in demonstrating RIRE between human macrophage U937 cells irradiated by α particles and non-irradiated HL-7702 hepatocyte cells upon co-culturing [49]. The authors further established that cyclic adenosine monophosphate (cAMP) was involved in the RIRE, which was communicated from the bystander cells to the irradiated cells through a membrane signaling pathway. When the U937 cells were irradiated, their cAMP levels decreased but were compensated by cAMP communicated from the bystander cells. Based on these findings, He et al. hypothesized that RIRE was mediated by the communicated cAMP [49]. In relation, Lam et al. [50,51] commented that the participation of cAMP was consistent with the activation of nuclear factor kappa B (NF-κB) pathway in RIRE (see the following subsection).

#### 2.4.2. Involvement of Activation of Nuclear Factor Kappa B (NF-κB) Pathway

As described in Section 2.3 above, Lam et al. [50,51] succeeded in demonstrating RIRE between α-particle-irradiated and non-irradiated human cervical cancer HeLa cells (i.e., between the same cell line) and between α-particle-irradiated and non-irradiated mouse embryo fibroblast NIH/3T3 cells (i.e., between the same cell line), either through (i) co-culturing the irradiated and non-irradiated cells, or (ii) treatment of irradiated cells with the medium having conditioned the non-irradiated counterparts which had in turn previously partnered with the corresponding irradiated cells. The authors further established from the co-culture experiments that the NF-κB pathway in irradiated cells was activated to produce the observed RIRE. The authors commented that activation of the NF-κB pathway in RIRE was in accordance with previous observations: (a) RIRE was mediated by soluble factors despite the presence of GJIC [44]; (b) RIRE was mediated by cAMP communicated from the bystander cells to the irradiated cells [49] (see the previous subsection); and (c) RIRE was accompanied by decreased levels of reactive oxygen species (ROS) in the irradiated cells [43]. Lam et al. proposed tumor necrosis factor-α (TNF-α) as a candidate that could activate the NF-κB pathway in irradiated cells, which, however, did not exclude other soluble molecules with a comparable function [50,51]. In fact, Kong et al. [34] found that interleukin 6 (IL-6) participated in communicating RIRE in their experiments (see Section 2.4.4 below).

#### 2.4.3. Involvement of NF-κB and COX-2 Signaling Pathways

As described in Section 2.3 above, Matsuya et al. revealed RIRE between normal human lung cells (either WI-38 fibroblasts or HBEC-3KT bronchial epithelial cells) within a region of the cell population irradiated non-uniformly with β-particles and γ-ray photons from a ^137^Cs- and ^134^Cs-bearing microparticle [46]. The two different normal human lung cells were studied in two sets of experiments (only a single cell type was employed in each set of experiment). Subsequently, Matsuya et al. reported that activation of the NF-κB p65 pathway signified the occurrence of RIRE [55]. Interestingly, however, significant dual activation of the NF-κB p65 and COX-2 pathways signified the occurrence of RIBE instead of RIRE [55].

#### 2.4.4. Induction of Autophagy and Interleukin 6 (IL-6) Secretion in Bystander Cells

As described in Section 2.3 above, Kong et al. [34] succeeded in demonstrating RIRE between X-ray-irradiated and non-irradiated human cervical cancer HeLa cells (i.e., same cell line). The authors hypothesized that RIRE was triggered by bystander factors sent from irradiated cells, which prompted autophagy and activated the signal transducer and activator of transcription 3 (STAT3) in the bystander cells to secrete IL-6 to activate the NF-κB pathway in irradiated cells. As per the GRS described in Section 2.2 above, Kong et al. [34] conjectured that RIRE could be interpreted as the outcome from metabolic cooperation, in that the bystander cells were elicited by the stressed cells to promote survival and growth of the latter.

Kong et al. [34] reported that without the bystander non-irradiated cells, autophagy was induced in the irradiated cells, which was hypothesized to contribute molecules needed for cell-repair enhancement. On the other hand, however, when the irradiated cells were partnered with bystander non-irradiated cells, autophagy was induced in the bystander cells, while at the same time autophagy accumulation in irradiated cells was reduced, which insinuated that the irradiated cells were aided through autophagy induction in the bystander cells. Interestingly, if autophagy was already pre-induced in the bystander cells before partnering with the irradiated cells, the RIBE exhibited in bystander cells diminished, while at the same time autophagy accumulation in irradiated cells was further reduced, and RIRE was further enhanced. Kong et al. further testified that IL-6 was secreted by bystander cells upon being cultured in the medium which had previously conditioned the irradiated cells, particularly so if autophagy had been pre-induced in the bystander cells [34]. These results demonstrated that IL-6 could initiate mitigation of damages induced in the irradiated cells.

As described in Section 2.2 above, based on the resemblance between RIRE and “metabolic cooperation”, Kong et al. proposed a GRS for RIRE and metabolic cooperation, which involved “adjudged stressed cells” and “adjudged bystander cells” in a way that the “adjudged bystander cells” would be elicited by the “adjudged stressed cells” to promote survival and growth of the latter [34]. “Metabolic cooperation” could be found in certain tumor microenvironments, e.g., when nutrient supply to the cancer cells was limited, neighboring normal cells would be elicited to provide nutrients to the cancer cells to promote survival and growth of the latter [36,37,38,39,40]. In analogy, RIRE could be found in a system consisting of irradiated and non-irradiated cells, e.g., when a required molecule to activate the NF-κB pathway (such as IL-6) in the irradiated cells to promote cell repair was limited, neighboring non-irradiated cells would be elicited to provide the molecule to the irradiated cells to promote cell repair of the latter [34]. Kong et al. also attempted to explain the occurrence of RIRE and BBR through the GRS between the “adjudged stressed cells” and “adjudged bystander cells” [34].

#### 2.4.5. Involvement of a Poly (ADP-Ribose) Polymerase 1 (PARP1)–NF-κB Positive Feedback Loop

As described in Section 2.3 above, Pathikonda et al. further confirmed RIRE between non-irradiated HeLa, MCF7, CNE-2, and HCT116 cells, and their corresponding partnered counterparts (same cell lines) irradiated with 200 kV X-rays through a 2 mm Al filter (X-RAD 320 Irradiator, Precision X-ray Inc., North Branford, CT, USA), through treatment of irradiated cells with the medium having conditioned the non-irradiated counterparts which had in turn previously partnered with the corresponding irradiated cells [47]. The authors further established that poly (ADP-ribose) polymerase 1 (PARP1) was involved in RIRE transcriptionally and translationally and that PARP1 and NF-κB were involved in the positive feedback loop transcriptionally and translationally.

#### 2.4.6. Involvement of Nitric Oxide (NO)

It was proven that nitric oxide (NO) could stimulate or inhibit NF-κB activity [56,57,58], while mechanisms established for NO-mediated RIBE [59,60,61,62,63,64,65,66] would also participate in RIRE. These previous results suggested that modulation of NF-κB activities could be dictated by the cell type and experimental conditions, and in particular, by the local concentrations of NO [67] or its redox end products [68], which could then control the occurrence of RIRE.

#### 2.4.7. Scheme for Activation of NF-κB Pathway in RIRE

**Figure 2:**
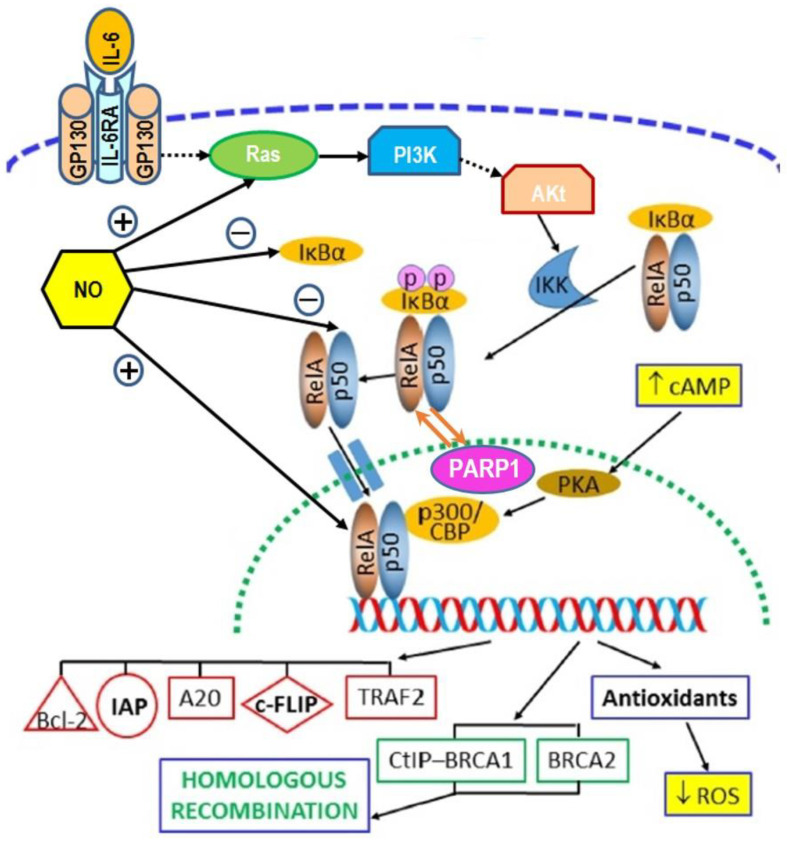
Summary of mechanisms and chemical messengers involved in RIRE: blue dashed line: cell membrane; green dotted line: nuclear envelope; dotted arrows: multiple steps involved. IL-6: interleukin 6; IL-6RA: interleukin 6 receptor alpha; RelA (p65) and p50: NF-κB family members (chosen for illustration here); NF-κB: nuclear factor κB; PARP1: poly (ADP-ribose) polymerase 1; cAMP: cyclic adenosine monophosphate; ROS: reactive oxygen species; NO: nitric oxide; GP130: glycoprotein 130; PI3K: phosphatidylinositol 3-kinase; Akt: protein kinase B (PKB); IKK: IκB-kinase; PKA: protein kinase A; CBP: CREB-binding protein; CREB: bcl-2: B-cell lymphoma 2 protein; IAP: inhibitors of apoptosis protein; A20: A20 zinc finger protein; c-FLIP: Cellular FLICE-like inhibitory protein; FLICE: FADD-like interleukin-1β-converting enzyme; FADD: Fas-associated protein with death domain; TRAF2: TNF receptor-associated factor 2; CtIP: C-terminal binding protein (CtBP)-interacting protein; BRCA1: breast cancer type 1 susceptibility protein; BRCA2: breast cancer type 2 susceptibility protein. (Adopted from ref. [20]; permission to reuse obtained from Elsevier).

Figure 2 presents the schematic diagram adopted from ref. [20] (with permission from Elsevier to reuse), which illustrates the activation of the NF-κB pathway by IL-6 in RIRE. Figure 2 also incorporates results from previous research on RIRE, namely, enhanced level of cAMP [49] and reduced level of ROS [43] in irradiated cells, as well as participation of NO [56,57,58,59,60,61,62,63,64,65,66]. The prototypical heterodimer with RelA (p65) and p50, which were NF-κB family members, were utilized for illustration.

### 2.5. Mechanisms Underlying Bilateral Bystander Response (BBR)

As described in Section 2.2 above, Fu et al. in 2016 reported the bilateral bystander response (BBR) where the radiobiological effects were exacerbated in the irradiated Beas-2B cells upon having co-cultured with non-irradiated U937 cells [32,33]. The mechanisms proposed for the BBR in their experiments included (a) activation of mitogen-activated protein kinases (MAPKs) and NF-κB pathways in the bystander U937 cells [32], and (b) upregulation of TNF-α and interleukin 8 (IL-8) in the bystander U937 cells, which were relayed on the activated extracellular signal–regulated kinases (ERKs) and p38 pathways in the irradiated Beas-2B cells and were also caused by the activated NF-κB pathway in the U937 cells [33].

## 3. Unique Features of Microbeam Radiations

In Section 3.1 below, the unique features provided by microbeam radiations for research on RIRE will first be reviewed, which includes their small beam size, facilitation of cell cultures with mixed targeted and non-targeted cells, well-defined irradiation areas, and delivery of ultra-high local doses and dose rates. Previous studies on RIRE using microbeam irradiation systems at NIRS and KEK of Japan will then be reviewed in Section 3.2 and Section 3.3, respectively.

### 3.1. Unique Features of Microbeam Radiations

#### 3.1.1. Small Beam Size to Target Individual Cells and Individual Cell Nuclei

Thanks to the small beam size employed for microbeam irradiations, individual cells and their nuclei could be irradiated without affecting other non-targeted cells or non-targeted parts of the targeted cells. For example, the beam diameter for the proton microbeams delivered by SPICE at NIRS was ~2 μm [69]. It is commonly established that most mammalian cells have a diameter between 10 to 100 µm, and their nuclei have an average diameter of ~6 µm, so the microbeams provide a convenient way to study RIRE, particularly if the targeted and non-targeted cells are mixed in a co-culture during irradiation to mimic a tumor environment or to provide geometric information on propagation of bystander signals (see Section 3.1.2 below). Furthermore, irradiating only the targeted cells can be achieved by directing the microbeam radiation towards those cells which exhibit fluorescence. The fluorescent images of the fluorescent targeted cell nuclei were captured by the online microscope system attached to a microbeam irradiation system, and the center positions of those cell nuclei were output as Cartesian coordinates. A specified amount of microbeam radiation was then directed towards each position according to its Cartesian coordinates [44,52,53].

#### 3.1.2. Mixing of Co-Cultured Targeted and Non-Targeted Cells

The small beam size to target individual cells and individual cell nuclei described above in Section 3.1.1 facilitated research with cell cultures consisting of a mixture of co-cultured targeted and non-targeted cells, which mimicked realistic tumor environments, as the non-targeted cells in the mixed co-culture would not be affected by the small microbeam size. Furthermore, when compared to the more traditional methods in studying RIRE such as the membrane inserts setup (e.g., ref. [14]) or the conditioned medium protocol (e.g., ref. [51]), the microbeam irradiation approach allowed cells in physical contact to develop gap junctions and allowed the irradiated and non-irradiated cells to stay in a common medium where soluble factors could freely move and diffuse. This provision can then yield results more directly relevant to radiation therapy [44,52,53]. The mixture of co-cultured targeted and non-targeted cells also yielded realistic geometric information on propagation of bystander signals [44].

As examples of exploiting such advantages of microbeam radiations in studying RIRE, it was possible to irradiate selected cells in a monoculture (e.g., of A549 cells) or to irradiate only one kind of cells in a co-culture (e.g., of A549 and WI38 cells) through prior fluorescent labeling of the targeted cells [44] or through constructing and using targeted cells that stably expressed GFP-tagged proteins [52]. Similarly, it was also possible to perform experiments on co-cultured CSCs and NSCCs [53].

#### 3.1.3. Well-Defined Irradiation Areas

The small beam size of microbeam radiations can also facilitate research which requires well-defined irradiation areas. An example is the study on the “radiation-induced field size effect” (RIFSE). As described and discussed in more details in Section 3.2 below, Ojima et al. utilized the 5.35 keV X-ray microbeam provided by the Photon Factory at KEK (Tsukuba, Ibaraki, Japan) to irradiate MRC-5 cells with various field sizes of 0.02, 0.09, 0.81, and 1.89 mm^2^ [17]. Ojima et al. also substantiated that energy deposition from the microbeam X-ray outside the beam area was negligible since the photoelectric effect was almost the exclusive interaction between the 5.35 keV X-rays and biological samples, from which the photoelectrons had a range of only ~0.8 μm [17].

#### 3.1.4. Delivery of Ultra-High Local Doses and Dose Rates

Ultra-high local doses and dose rates can be delivered to targeted cells, although these might not have been the original objectives of research works that made use of microbeam radiation. However, interest in the so-called FLASH effect, which has been commonly considered to correspond to mean dose rates of ≳40 Gy s^−1^ (beyond the clinical standard of the order of Gy min^−1^ [21,29]), has remarkably intensified in recent years (see Section 4.5 below.

As described in Section 2.3 above, the SPICE at NIRS provided protons with an energy ~3.4 MeV in a beam with a diameter ~2 μm, and could deliver 500 protons per cell in 0.05 s. The doses and dose rates of microbeam protons delivered to A549 human lung cancer cells and WI38 human lung normal fibroblasts, which were employed by Desai et al. [44] and Kobayashi et al. [52] in their studies (see below), using SPICE at NIRS were estimated by Kobayashi et al. [52]. However, Kobayashi et al. also cautioned about the inevitable uncertainties due to the ambiguous definition of the dose, namely, whether the mass involved should be (i) that of the entire cell nucleus, or (ii) that of the portion of the cell nucleus traversed by the microbeam protons only (with microbeam diameter of 2 μm) [52]. For method (i), i.e., using the mass of the entire cell nucleus to calculate the doses, the average absorbed doses in the cell nuclei of A549 and WI38 cells per 500 protons were estimated as 5.1 and 3.8 Gy, respectively. Taking the dose delivery time as 0.05 s, the mean dose rates would then be 102 and 76 Gy s^−1^, respectively. For method (ii), the average absorbed dose in the cell nuclei of A549 cells per 500 protons became 296 Gy, and the corresponding dose rate became 5920 or ~6 × 10^3^ Gy s^−1^, i.e., larger by a factor of 58! Interestingly, no matter whether it was 102 or 6 × 10^3^ Gy s^−1^, the dose rates fell into the FLASH effect region.

Similarly, Liu et al. also used SPICE at NIRS to irradiate nonstem-like cancer cells (NSCCs) and cancer stem-like cells (CSCs) of the human fibrosarcoma HT1080 cell line in their studies and estimated the dose absorbed by the cell nuclei to be around 0.92 Gy per irradiated proton. Considering the delivery of 500 protons per cell in 0.05 s, the dose rate could reach 9 × 10^3^ Gy s^−1^, which again fell into the FLASH effect region. In contrast, however, at the Photon Factory, KEK, the generated microbeam X-rays typically had a photon fluence of ~ 2 × 10^8^ photons/1.89 mm^2^/s or 10^14^ photons m^−2^s^−1^, which was equivalent to a dose of 0.25 Gy s^−1^ [17] and was far below the FLASH effect region.

Whether microbeam radiations could also provide a platform to study the FLASH effect or whether the results on RIRE obtained from previous or future microbeam experiments would represent the resultant effect from (RIRE + FLASH effect) certainly deserves more attention and future research effort.

### 3.2. RIRE Studies Using SPICE at NIRS

To date, all experiments demonstrating microbeam-proton-induced RIRE have been carried out using SPICE at NIRS, Chiba, Japan. There were three in vitro studies, i.e., two on RIRE between lung adenocarcinoma (A549) cells and human lung normal fibroblast (WI38) cells [44,52] and one on RIRE between NSCCs and CSCs in human fibrosarcoma HT1080 [53], which will be reviewed in Section 3.2.1 to Section 3.2.3. The in vivo study on RIRE between cells in zebrafish embryos (*Danio rerio*) at the two-cell stage will be reviewed in Section 3.2.4.

#### 3.2.1. RIRE between Lung Adenocarcinoma (A549) Cells and Human Lung Normal Fibroblast (WI38) Cells [44]

**Figure 3:**
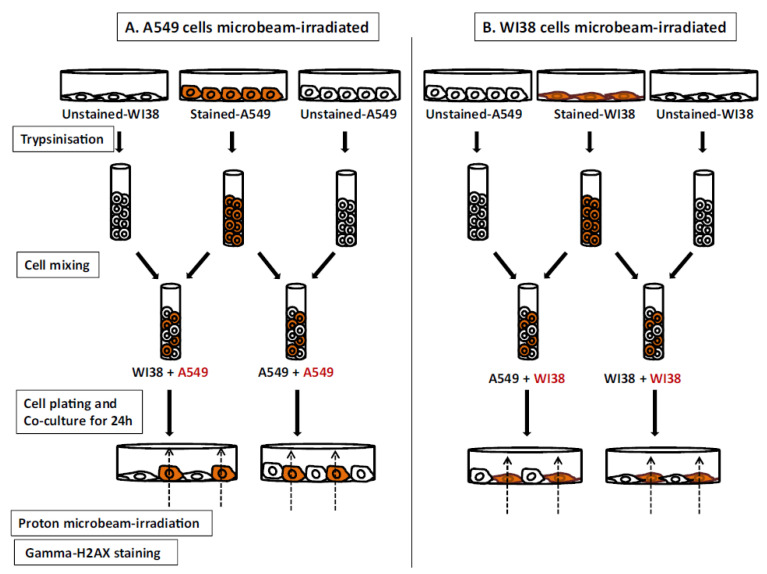
Experimental scheme employed in the work of Desai et al. [44]. In the beginning, the chosen targeted cells (either (**A**). A549 cells or (**B**). WI38 cells) and non-targeted cells were cultured in separate dishes. One day before the microbeam irradiation, the chosen targeted cells were labeled with cell tracker orange (CTO) in their own dishes and were then mixed with the unlabeled non-targeted cells in a 1:1 ratio to form a co-culture in the “co-culture dish” for the subsequent microbeam-proton irradiations. (Adopted from Desai et al. [44]; permission to reuse obtained from Elsevier).

Desai et al. studied RIRE between microbeam-proton-irradiated A549 or WI38 cells with co-cultured bystander A549 or WI38 cells, employing γ-H2AX foci fluorescence intensity per nucleus as the biological end point (which surrogated the number of DNA double-strand breaks, i.e., DSBs) [44]. In the beginning, the chosen targeted cells and non-targeted cells were cultured in separate dishes. One day before the microbeam irradiation, the chosen targeted cells were labeled with cell tracker orange (CTO) in their own dishes and were then mixed with the unlabeled non-targeted cells in a 1:1 ratio to form a co-culture in the “co-culture dish” for the subsequent microbeam-proton irradiations (with the experimental scheme shown in Figure 3). CTO is a non-leaky fluorescent dye in that it will not pass from one cell to another.

All the cells in the co-culture dish, regardless of whether labeled or unlabeled with CTO, were stained with Hoechst 30 min before irradiation. The nuclei of the chosen targeted cells, i.e., those labeled with CTO, were then each irradiated with 500 protons. For the experiments to assess the involvement of GJIC, the cells were treated with lindane before irradiation to inhibit GJIC.

Desai et al. confirmed the emergence of RIRE between bystander WI38 cells and irradiated A549 cells, which was shown to be much stronger than the RIRE between bystander A549 cells and irradiated A549 cells [44]. The RIRE between bystander WI38 cells and irradiated A549 cells was further proved to be independent of GJIC (in contrast to their finding that the RIBE between bystander A549 cells and irradiated A549 cells depended on GJIC). Based on this observation, Desai et al. speculated that soluble factors secreted by WI38 cells played a role in the RIRE between bystander WI38 cells and irradiated A549 cells [44]. On the other hand, the authors confirmed that no RIRE occurred for irradiated WI38 cells (regardless of whether the bystander cells were A549 or WI38 cells). The possible reasons behind the differences in the RIRE in irradiated A549 and WI38 cells, namely, the presence in the former and absence in the latter, were not discussed by Desai et al. [44].

Nevertheless, the authors highlighted the different geometries of the two types of cells which led to dissimilar average absorbed doses upon irradiation with the same number (500) of protons, namely, 5.1 Gy and 3.8 Gy for A549 and WI38 nucleus, respectively. Such difference was exploited to explain their observations: (1) DNA damages (surrogated by the intensity of γ-H2AX foci per irradiated cell nucleus) in irradiated WI38 cell nuclei were significantly less significant than those in irradiated A549 cell nuclei upon irradiation with the same number of protons, and (2) irradiation with more protons (200 protons) was needed to induce DSBs in WI38 cells than in A549 cells (50 protons). Whether such reasoning can be further utilized to explain the different RIRE occurrence is worth further deliberation and more future research work (see also Section 4.2 and Section 4.3 for further discussion).

Desai et al. also reported the detection of RIBE in bystander A549 cells but not in bystander WI38 cells after they were co-cultured with irradiated A549 cells, while RIBE was not detected in either bystander A549 or WI38 cells after they were co-cultured with irradiated WI38 cells [44]. This might appear intriguing since RIRE was detected between bystander WI38 cells and irradiated A549 cells. Nevertheless, this observation was in line with the previous finding by Kong et al. that an enhanced RIRE in the irradiated cells might not necessarily require stronger RIBE displayed in the corresponding bystander cells [34]. Specifically, Kong et al. reported that if autophagy was pre-induced in the bystander cells prior to partnering with the irradiated cells, the RIBE displayed in the bystander cells weakened, while at the same time the RIRE delivered to the irradiated cells was enhanced [34]. The absence of RIBE in bystander A549 or WI38 cells upon co-culturing with irradiated WI38 cells, despite the presence of RIRE between bystander WI38 cells and irradiated A549 cells, was also commensurate with the previous finding by Kong et al. that unirradiated zebrafish embryos needed the mediated nitric oxide (NO), but without developing NO-induced damages themselves, to exert RIRE on their partnered irradiated zebrafish embryos [70]. Lam et al. proposed that the bystander signals secreted by the irradiated cells, which led to the subsequent secretion of rescue signals from the bystander cells, did not necessarily lead to detectable damage in the bystander cells [15].

Last but not least, Desai et al. also established that RIRE between bystander WI38 cells and irradiated A549 cells was independent of GJIC, although the authors proved that RIBE between irradiated and bystander A549 cells was mediated through GJIC [44]. Whether the non-involvement of GJIC in the RIRE between bystander WI38 cells and irradiated A549 cells was accountable (at least partially) for (i) the much stronger RIRE between bystander WI38 cells and irradiated A549 cells, when compared to the RIRE between bystander and irradiated A549 cells; and/or (ii) the absence of RIBE in bystander A549 or WI38 cells upon co-culturing with irradiated WI38 cells, despite the presence of RIRE between bystander WI38 cells and irradiated A549 cells, would certainly be enlightening and deserve further investigations.

#### 3.2.2. RIRE between Lung Adenocarcinoma (A549) Cells and Human Lung Normal Fibroblast (WI38) Cells [52]

Kobayashi et al. [52] followed up on the studies of Desai et al. [44] on RIRE between A549 cells and WI38 cells with revised experimental setup, namely, (i) a revised method to generate fluorescence from the targeted cells for microbeam proton irradiation, and (ii) a revised arrangement of cells in the irradiation dish for additional assessment on the propagation of RIBE signals between the irradiated A549 cells and bystander A549 cells or bystander WI38 cells. The γ-H2AX level in the cell nuclei was similarly employed as the biological end point but was detected over a longer period for up to 24 h post-irradiation by Kobayashi et al. [52].

As regards the generation of fluorescence from the targeted cells, instead of labeling them with CTO [44], Kobayashi et al. [52] constructed the A549-GFP cell line by transfecting the pBOSH2BGFP vector into A549 cells to stably express the GFP-tagged histone H2B fusion protein. In the study of Kobayashi et al., only the targeted cells that generated fluorescence and were recognized by the online microscope system attached to microbeam irradiation system, i.e., only A549-GFP cells but not the A549 cells or WI38 cells were irradiated [52].

**Figure 4:**
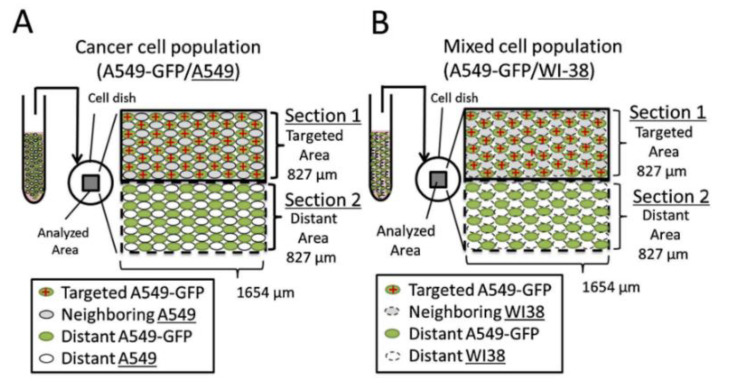
Experimental scheme employed in the work of Kobayashi et al. [52]. Each cell dish was divided into two halves, with one half (Section 1) containing both targeted and non-targeted cells while the other half (Section 2) contained only non-targeted cells. Only A549-GFP cells (stably expressing H2BGFP) in Section 1 were targeted in the experiments. There were thus three different types of cells in the co-cultures, namely, (i) targeted A549-GFP cells in Section 1, each of which was irradiated with 500 protons; (ii) non-targeted A549 cells (in the cancer cell population (**A**)) or non-targeted WI38 cells (in the mixed cell population (**B**)), both of which were “neighboring” and having direct contact with the “targeted” A549-GFP cells in (i); and (iii) non-targeted A549-GFP cells, A549 cells, or WI38 cells in Section 2, which were also referred to as the “distant” non-targeted A549-GFP, A549, or WI38 cells, respectively, and were not in direct contact with the “targeted” A549-GFP cells in (i). (Adopted from ref. [52]; permission to reuse obtained from Elsevier).

Regarding the revised arrangement of cells in the irradiation dish, as illustrated in Figure 4, each cell dish for irradiation was separated into two halves, with one half (referred to as Section 1) containing both targeted cells (A549-GFP cells) and non-targeted cells (A549 cells or WI38 cells), while the other half (referred to as Section 2) contained only non-targeted cells. Three different types of cells were present in the co-cultures, namely, (i) targeted A549-GFP cells in Section 1, each of which would be irradiated with 500 protons; (ii) non-targeted A549 cells (in the cancer cell population [case A]) or non-targeted WI38 cells (in the mixed cell population [case B]), both of which were “neighboring” and having direct contact with the “targeted” A549-GFP cells in (i); and (iii) non-targeted A549-GFP cells, A549 cells, or WI38 cells in Section 2, which were also referred to as the “distant” non-targeted A549-GFP, A549, or WI38 cells, respectively, and were not in direct contact with the “targeted” A549-GFP cells in (i). Simply put, categories (ii) and (iii) could also be described as “neighboring” and “distant” bystander cells, respectively. In principle, the “neighboring” bystander cells (ii) in Section 1 of the cell dish would be able to form GJs with “targeted” A549-GFP cells (i).

Kobayashi et al. confirmed the occurrence of RIRE between bystander WI38 cells and irradiated A549-GFP cells [52]. The observation was expected because Desai et al. also previously confirmed the occurrence of RIRE between bystander WI38 cells and irradiated A549 cells [44]. Moreover, Kobayashi et al. revealed that the RIRE between bystander WI38 cells and irradiated A549-GFP cells was much stronger than that between bystander A549 cells and irradiated A549-GFP cells at 4 and 8 h post-irradiation, and this pattern was maintained over a period for up to 24 h post-irradiation [52]. These results were in line with the previous conclusion made by Desai et al. that the RIRE between bystander WI38 cells and irradiated A549 cells was much stronger than that between bystander A549 cells and irradiated A549 cells [44].

#### 3.2.3. RIRE between Nonstem-like Cells and Stem-like Cells in Human Fibrosarcoma HT1080 [53]

Liu et al. [53] reported the occurrence of RIRE between irradiated nonstem-like cancer cells (NSCCs) and non-irradiated cancer stem-like cells (CSCs) of the human fibrosarcoma HT1080 cell line. The authors investigated possible RIRE in all four different combinations of the two cell types, using the level of 53BP1 accumulation as the biological end point. The team co-cultured CSCs and NSCCs and chose a specific cell type to be irradiated with microbeam protons, which was set at 15% of the cell co-culture. The nuclei of the targeted cells were first stained with CTO to make them recognizable by the microscopic system, which would then be irradiated with 20, 50, or 200 protons. To assess the significance of RIRE, the relative fluorescence unit (RFU) of nucleus 53BP1 foci were measured, where a higher RFU of focus meant more accumulation of 53BP1. The RFU of focus in targeted NSCCs was significantly reduced upon having co-cultured with bystander CSCs when compared to the case for having co-cultured with bystander NSCCs (1.35 ± 0.37 folds cf. 2.25 ± 0.40 folds; *p* < 0.01). In contrast, the RFU of foci in targeted CSCs were not significantly different upon having co-cultured with bystander CSCs or NSCCs (2.19 ± 0.29 folds cf. 2.20 ± 0.34 folds; *p* = 0.85). The authors concluded that bystander CSCs could mitigate damages in targeted NSCCs but not in targeted CSCs, and bystander NSCCs did not mitigate damages in targeted CSCs or NSCCs.

#### 3.2.4. RIRE between Cells in Zebrafish Embryos (*Danio rerio*) Irradiated at the Two-Cell Stage [54]

Choi et al. provided evidence on the occurrence of RIRE between microbeam-proton-irradiated cells and their non-irradiated counterparts in zebrafish embryos (*Danio rerio*) irradiated at the two-cell stage at 0.75 h post fertilization (hpf), employing the number of apoptotic signals per zebrafish embryo quantified at 25 hpf through terminal dUTP transferase-mediated nick end-labeling (TUNEL) assay as the biological end point [54].

Choi et al. employed SPICE at NIRS to irradiate dechorionated zebrafish embryos (*Danio rerio*) at the two-cell stage by microbeam protons, with a control on the irradiation spots [54]. To ensure sufficient numbers of synchronized two-cell stage zebrafish embryos, adult zebrafish were maintained with a 14/10 h light-dark cycle to maintain a reliable production of embryos. At the start of the 14-h photo period, a specially designed plastic container [71] was deployed to collect the embryos within a period no longer than 30 min to ensure synchronization of the embryos. The collected embryos were then dechorionated and irradiated at the two-cell stage [72].

A specially designed irradiation setup was constructed to accommodate the zebrafish embryos for their irradiation, which consisted of a dish consisting of a Si_3_N_4_ plate and a steel ring for mounting a Mylar film as the substrate of the embryos. The dechorionated embryos were placed in the irradiation setup with the cells oriented towards the Mylar film. After the 3.4-MeV protons passed through the Si_3_N_4_ exit window, the Mylar film, and the air gap between the two, their energies would drop to 3.37 MeV when they reached the target cells. In separate experiments, one cell or both cells of the two-cell stage embryos were irradiated with 10, 20, 40, 50, 80, 100, 160, 200, 300, and 2000 protons, which were referred to as (×1) case (i.e., one cell of the two-cell stage embryos was irradiated) and (×2) case (i.e., both cells of the two-cell stage embryos were irradiated), respectively. The irradiated embryos were then returned to the incubator until 25 hpf, when their apoptotic signals were evaluated through TUNEL assay [54].

Choi et al. computed the conversion into absorbed dose *D* as 0.15 mGy per proton through *D* = *E*/*M*, where *E* was the energy of incident protons, and *M* was the estimated mass of a cell of the two-cell stage zebrafish embryo [54]. However, as mentioned in Section 3.1.2 above, Kobayashi et al. cautioned about the inevitable ambiguous definition of dose in microbeam irradiation experiment in that the mass involved could be that of the biological material traversed by the microbeam protons only (with microbeam diameter of 2 μm) [52]. The adoption of this definition would increase the conversion to around 0.6 Gy per proton.

After recording the numbers of apoptotic signals on the 25 hpf zebrafish embryos, Choi et al. computed the normalized averages with references to the averages of the corresponding control samples obtained with sham irradiations [54]. Normalization was required for more accurate results and interpretation as the average numbers of apoptotic signals recorded for control samples changed for different experiments. Table 2 in the current review paper has shown extracts of their data on (a) the normalized values (of the numbers of apoptotic signals), and (b) comparisons of these values for the (×1) and (×2) cases with the control samples. Choi et al. computed the *p* values from *t*-tests and referred differences corresponding to *p* ≤ 0.05 as statistically significant, while those corresponding to *p* > 0.05 and ≤ 0.07 were referred to as “marginally significant” [54]. The authors also remarked that the apoptotic signals from the control samples stemmed from spontaneous or endogenous damages.

From these data, Choi et al. identified significant differences in the dose responses for the (×1) and (×2) cases [54]. Simply put, most of the (×1) cases led to apoptotic signals close to those of the controls (particularly when the number of irradiated protons was <160), while quite a number of the (×2) cases led to apoptotic signals significantly different from those of the controls. Choi et al. provided a comprehensive discussion on such significant differences between the (×1) and (×2) cases and attributed such differences to the more efficient repair of DNA DSBs in the (×1) cases to RIRE [54]. Interested readers are referred to their paper. In association, the “triphasic” dose response for the (×2) case was similar to the responses observed in 5 hpf zebrafish embryos where RIRE was less effective [73,74].

### 3.3. RIRE Studies Using Synchrotron X-ray Microbeam Irradiation System at the BL-27B Station in the Photon Factory, KEK [17]

Ojima et al. envisaged the occurrence of RIRE between microbeam-X-ray-irradiated primary normal human lung fibroblasts, MRC-5, with their co-cultured non-irradiated counterparts, employing the number of 53BP1 (p53 Binding Protein 1) foci per cell as the biological end point (which surrogated the number of DSBs in cell nuclei), when the authors were studying the RIFSE [17]. The authors employed defined-size monochromatic 5.35 keV X-rays supplied by the synchrotron X-ray microbeam irradiation system at the BL-27B station in the Photon Factory of KEK (Tsukuba, Ibaraki, Japan) to irradiate MRC-5 cell populations cultured on cover glasses accommodated in 35-mm dishes, with various field sizes, namely, 0.02, 0.09, 0.81, and 1.89 mm^2^ [17]. The grid lines on the cover glasses allowed logging the positions of the irradiated cells under a microscope. Before irradiation of the cells, the beam image was captured to enable alignment between its center and the center of the cell samples. The cell samples were then microbeam-X-ray-irradiated with a dose of 1 Gy. The authors further substantiated that energy deposition from the microbeam X-ray outside the beam area was negligible, since photoelectric effect was almost the exclusive interaction between the 5.35-keV X-rays and biological samples, from which the photoelectrons had a range of only ~0.8 μm [17]. The numbers of 53BP1 foci per cell were analyzed for up to 48 h post-irradiation.

Ojima et al. reported that the number of 53BP1 foci per cell generally increased with the field size but saturated at the field size of 0.81 mm^2^ for all measured time points up to 48 h post-irradiation. Based on these findings, the authors concluded that RIFSE was confirmed in X-ray-irradiated MRC-5 cells. Specifically, the authors explained RIFSE in terms of RIRE where the rescue signal released from the out-of-field non-irradiated cells enhanced the repair of DNA damages in the in-field irradiated cells [17]. The authors further explained the saturation of the RIFSE at the field size of 0.81 mm^2^ through related findings for RIRE by Lam et al. [51] where the RIRE (a) depended on the relative abundance of bystander cells, i.e., the ratio *R* of number of unirradiated bystander cells/number of irradiated cells, and (b) displayed a saturation (smaller change) in the response for large *R* values. Furthermore, Ojima et al. [17] revealed that the number of 53BP1 foci per cell was lower in the irradiated cells close to the border of the in-field area, i.e., those irradiated cells next to the out-of-field area. The authors attributed this observation to the time needed for diffusion of bystander signals from the irradiated cells to the non-irradiated bystander cells [75].

## 4. Discussion

The main objective of the current paper was to provide a review on the results on radiation-induced rescue effect (RIRE) between targeted irradiated cells and non-irradiated bystander cells obtained from previous microbeam experiments. As described in the beginning of Section 3, microbeam irradiations offered some unique opportunities for RIRE research. Many of the previous findings related to RIRE reviewed in Section 3 were made possible thanks to these unique opportunities provided by various microbeam irradiation systems. Further discussion on these findings and some thoughts on future priorities and directions of research on RIRE exploiting the unique features of microbeam radiations will be presented in this section.

### 4.1. Mechanisms Underlying RIRE

As reviewed in Section 2.4, the mechanisms underlying RIRE included the activation of the NF-κB pathway in the irradiated cells [50,51]. This mechanism encompassed the other findings that RIRE involved (a) participation of PARP1 and the PARP1–NF-κB positive feedback loop [47], (b) mediation of cAMP from bystander cells to irradiated cells through a membrane signaling pathway [49], (c) soluble factors in spite of the presence of gap junctions [44], (d) reduction in ROS levels in the irradiated cells [43], and (e) participation of NO [56,57,58,59,60,61,62,63,64,65,66] (see Section 2.4.7 and the scheme for activation of NF-κB pathway in RIRE shown in Figure 2). Activation of the NF-κB pathway was also subsequently incorporated into the mechanism proposed for RIRE by Kong et al. [34] that was first triggered by bystander factors sent from irradiated cells, which then prompted autophagy and activated STAT3 in the bystander cells to secrete IL-6 to activate the NF-κB pathway in the irradiated cells.

Apparently, a better understanding on the mechanisms underlying RIRE will allow better comprehension or prediction on treatment outcomes of radiotherapy procedures and will also enable development of drugs or alternative treatment procedures through mitigating or exploiting RIRE. In addition, Kong et al. [34] discussed the potential insights that could be gained if RIRE and metabolic cooperation had a common origin, where metabolic cooperation referred to the cross talk between cancer cells and non-transformed cells (see Section 2.2 above and Section 4.2 below). If the common origin could be verified, an alternative strategy to treat cancers involving metabolic cross talk, such as pancreatic cancers, would be inhibition of secretion of relevant bystander factors, while an alternative strategy to treat cancers involving RIRE could be inactivation of autophagy pathways in the bystander non-irradiated cells [34].

Interestingly, besides confirming that activation of the NF-κB pathway signified the occurrence of RIRE, Matsuya et al. further reported that significant dual activation of the NF-κB and COX-2 pathways signified the occurrence of RIBE, instead of RIRE [55]. Through identification of different activated pathways, upon non-uniformly irradiating a cell sample, Matsuya et al. succeeded in identifying regions in the irradiated cell sample which were dominated by RIRE and by RIBE [55]. It was remarked that this important result could help pinpoint the origin of the RIFSE [20] (see Section 4.5 below) and could also inspire a research direction in RIRE in general to ascertain the signaling pathways and chemical messengers involved in cell samples which had undergone different irradiation conditions, including non-uniform irradiations. Notably, there has been research into the application of microbeam radiation for spatially fractionated radiotherapy, the so-called microbeam radiotherapy (MRT) [76], from which lower toxicity to normal tissues has been demonstrated in a large number of animal studies. Apparently, a better understanding of the mechanisms underlying RIRE and the spatially fractionated non-uniform irradiation of MRT would be needed to figure out whether RIRE has played a role in this tissue sparing effect of MRT. Microbeam irradiation setups which exploited the small beam size and allowed cell cultures with mixed targeted and non-targeted cells and well-defined irradiation areas, such as those employed by Kobayashi et al. [52] and Ojima et al. [17], would be particularly useful to deliver the different irradiation conditions. Such projects would be good future priorities in the research on RIRE using microbeam radiations.

### 4.2. Generalized Rescue Scheme (GRS)

As summarized in Section 3.2 above, previous RIRE studies using microbeam protons included RIRE between A549 cells and WI38 cells [44,52] and RIRE between NSCCs and CSCs in human fibrosarcoma HT1080 [53]. An intriguing observation was that the occurrence or the strength of RIRE apparently depended on the cell type.

Under the “generalized rescue scheme” (GRS), the difference in RIRE in irradiated A549 cells and WI38 cells, namely presence in the former and absence in the latter [44,52], could be explained if the cell type dominated over the state of irradiation in adjudging the ultimate status of “adjudged stressed cells”. The differences in RIRE in irradiated NSCCs and CSCs, namely presence in the former and absence in the latter [53], could also be explained if the cell type dominated over the state of irradiation in adjudging the ultimate status of “adjudged stressed cells”, and if NSCCs and CSCs played the roles of “generalized stressed cells” and “generalized bystander cells”, respectively. The reasons underlying the roles played by NSCCs and CSCs are not yet understood, although peculiar features such as radioresistance were identified in various CSCs, which might be linked to their roles played in the GRS. For example, Diehn et al. revealed that subsets of CSCs in some tumors, similar to normal tissue stem cells, contain lower levels of ROS and stronger ROS defenses than their non-tumorigenic progeny, which might explain the tumor radioresistance [77].

One important extract from the above intriguing observations can be summarized as follows: a non-irradiated bystander cell type (denoted as type A here) can provide RIRE to an irradiated cell type (denoted as type B here) but cannot provide RIRE to another irradiated cell type (denoted as type C here; C and A can be the same). Regarding this observation, it would be interesting to study whether the rescue signals released from the non-irradiated bystander cell type A in the former case (where RIRE exists between non-irradiated bystander cell type A and irradiated cell type B) if collected and applied to the irradiated cell type C can lead to RIRE in the latter. In relation, a methodology for physical separation of the rescue signals from the bystander signals was developed by Lam et al. [51]. Such a study could provide further information on the mechanisms underlying RIRE among the cell types A, B, and C. The results from such studies might also help formulate strategies to control the strength of RIRE delivered to a selected irradiated cell type (type B) through introducing another irradiated cell type (type C) into the cell population to compete for the limited amount of rescue signals. The strategy, if proved successful, might provide options to minimize the undesirable rescue of cancer cells during radiotherapy.

An example is given here to elucidate the strategy by referring to the RIRE between WI38 and A549 cells described above, where there was strong RIRE between bystander WI38 cells and irradiated A549 cells but no RIRE between bystander and irradiated WI38 cells [44]. If the rescue signal released by non-irradiated WI38 cells when prompted by irradiated A549 cells could also provide RIRE to irradiated WI38 cells at the same time, it might then be feasible to control the strength of RIRE delivered to the irradiated A549 cells through introducing “irradiated” WI38 cells into the cell population to compete for the rescue signals.

An experiment employing a mixed cell co-culture on a slide glass could be proposed to verify the above strategy, in which WI38 cells are cultured on half on the slide and A549 cells are cultured on the other half. The strategy would be to irradiate a number of columns of WI38 cells along the border between the two cell types in addition to the irradiation of the targeted A549 cells. If rescue signals were released only from the non-irradiated WI38 cells, which would need to be confirmed, the irradiated WI38 cells would compete with the irradiated A549 cells for the rescue signals, thereby weakening the strength of RIRE delivered to the irradiated A549 cells. It would then be interesting to determine the relationship between the strength of RIRE delivered to the irradiated A549 cells with the number of irradiated WI38 cell columns. Microbeam irradiation setups which exploited the small beam size and well-defined irradiation areas, together with the techniques of rendering the targeted cells fluorescent to be irradiated with the microbeam radiation [44,52,53], would be particularly useful for determining this relationship.

In relation, competition of rescue signals in an irradiated cell population was first observed by Lam et al. who studied RIRE in HeLa and NIH/3T3 cells [51]. In particular, Lam et al. assessed the dependence of the strength of RIRE on the relative abundance of bystander cells. For irradiation of 2.5% of the cells, both cell lines developed statistically significant RIRE; however, for irradiation of 75% of the cells, only NIH/3T3 cells but not HeLa cells developed statistically significant RIRE [51]. More recently, as described in Section 3.3 above, Ojima et al. established that non-irradiated MRC-5 cells rescued their partnered counterparts within X-ray irradiated fields with sizes of 0.02, 0.09, 0.81, or 1.89 mm^2^, through demonstrating the RIFSE [17]. The authors also revealed that the strongest RIRE was delivered to irradiated cells within the smallest-size irradiation field. Most recently, as also described in Section 3.3 above, Matsuya et al. irradiated different percentages of HBEC3-KT cells in a culture flask, viz., 25%, 50%, 75%, and 100%, with X-rays and found strongest RIRE for irradiation of 25% of the cells [55]. In relation, Choi et al. reported RIRE between α-particle irradiated zebrafish embryos and bystander unirradiated zebrafish embryos and also found that the strength of RIRE increased when the number of bystander embryos increased from 10 to 30, while keeping the number of irradiated embryos at 10 [78].

### 4.3. Radiation Dose Response of RIRE

Yu [16] summarized some previous research results in the literature which might demonstrate the effects of radiation dose on RIRE. Chen et al. employed 20 or 40 cGy of alpha particles for their experiments and succeeded in demonstrating RIRE between NHLF cells [14]. Pereira et al. [45] employed 70 or 550 mGy of gamma ray photons from a ^137^Cs gamma irradiator for their experiments and succeeded in demonstrating RIRE between ZF4 cells. In contrast, Widel et al. [43] employed 2 or 4 Gy of 6 MV X-ray photons for their experiments but did not detect (although noting an indication of) RIRE in HCT116 cells. Apparently, the discrepancies could be explained by the different radiation doses used, but these could also be attributed to the different types of ionizing radiation and/or different cell lines.

In relation, Desai et al. attempted to explain their observation that the occurrence or the strength of RIRE depended on the cell type as described in Section 3.2.1 above through considering the differences in the absorbed radiation doses upon irradiation with the same number (500) of protons, namely, 5.1 Gy and 3.8 Gy for A549 and WI38 nucleus, respectively [44]. However, the brief literature review in the preceding paragraph showed that larger irradiation doses did not necessarily lead to more likely occurrence of RIRE.

As such, it is advocated that establishment of dose responses for RIRE is important for deciphering the phenomenon and is also a key to better understanding or predicting treatment outcomes of chosen radiotherapy procedures (e.g., refs. [15,16]). In relation, as highlighted in the discussion on the GRS in Section 4.2 above, the occurrence or the strength of RIRE depended on the “status” of the cells involved as well, e.g., whether the “cell type” or “state of irradiation” dominated in dictating the status of “adjudged stressed cells” or “adjudged bystander cells”. It is still not yet clear but is certainly relevant to look into whether or how the doses absorbed by the irradiated cells would affect their status. For example, the dependence of the cell status on the absorbed dose might help elucidate the reasons why NSCCs and CSCs played the roles of “adjudged stressed cells” and “adjudged bystander cells”, respectively, in the study of Liu et al. [53], which was speculated above to be related to peculiar features such as radioresistance identified in various CSCs. Towards the end of Section 4.2 above, a proposal to design strategies to control the strength of RIRE delivered to a selected cell type in a cell population which might provide options to minimize the undesirable rescue of cancer cells during radiotherapy. A crucial element in the strategy was the availability of cells which upon irradiation would only take up available rescue signals. At the same time, the cells would not release rescue signals. This would impose even more stringent requirements on the status of the cells and thus on the irradiation dose if the cell status is dose-dependent.

Furthermore, as discussed in Section 3.2.4 above and Section 4.5 below, the dose rates and dose delivery time of microbeam protons at SPICE of NIRS appeared to fall into the FLASH effect regime. As such, it would be important and interesting to examine the dose response where the radiobiological effects represent the resultant from the RIRE + FLASH effect.

### 4.4. Radiation-Induced Field Size Effect (RIFSE)

As described in Section 3.2 above, when Ojima et al. were studying RIFSE in MRC-5 cells irradiated by microbeam X-ray photons, they found evidence of RIRE between irradiated and non-irradiated cells [17]. Specifically, the authors attributed the RIFSE observed in their experiments to RIRE that boosted the repair of DNA damages in the in-field irradiated cells, which was triggered by the rescue signal released from the out-of-field non-irradiated cells.

Yu [20] recapped that RIFSE was fundamentally important for radiological protection consideration. For example, only average equivalent doses could be computed even for non-uniform irradiation when the effects from different field sizes were not taken into account, which might sometimes cause erroneous interpretation of results and conclusions. Yu [20] also reviewed the implications of RIFSE in different branches of medical physics, including, e.g., the effects of modulated fields in intensity-modulated radiation therapy (IMRT) and microbeam radiotherapy (MRT).

In fact, RIFSE was revealed as early as 1984 by Coggle et al. [18] and Peel et al. [19] who irradiated mouse skin and pig skin with broad-beam β particles with different irradiation field size. The first link between RIFSE and RIRE was provided by study of Lam et al. [51] who reported that the damage in the α-particle-irradiated NIH/3T3 cells and HeLa cells depended on the relative abundance between unirradiated cells and irradiated cells, or equivalently the size of the irradiated area. In 2012, Butterworth et al. irradiated different cell lines with intensity-modulated X-ray fields from an X-ray irradiator and detected larger survival response in human prostate cancer DU-145 cells for an in-field area proportion of 25% when compared to that for uniform exposure [79]. This was a strong evidence of the action of RIRE although the authors did not explicitly make reference to the effect [20]. It was noted that RIRE only showed up for the in-field area proportion of 25%, and not for in-field area proportions of 50% and 75%, further portrayed the impact of the relative abundance of bystander cells on RIRE [20].

More recently, as described in the beginning of this section, Ojima et al. observed RIFSE in their microbeam-X-ray irradiated MRC-5 cells [17]. To the best of our knowledge, this study was the first one that employed a microbeam radiation (microbeam X-rays in this case) to demonstrate RIFSE, which was then attributed to RIRE.

Most recently, Matsuya et al. examined the radiobiological responses of normal human lung cells (WI-38 fibroblasts and HBEC-3KT bronchial epithelial cells) upon uniform irradiation and local non-uniform irradiation [46,55]. The different sizes of irradiated areas due to the different irradiation scenarios could be viewed as different radiation field sizes. Matsuya et al. reported that beyond a certain absorbed dose, the non-uniform irradiation inflicted fewer damages in the cells than the uniform irradiation, which was attributed by the authors to RIRE (as a potential explanation) [46]. Importantly, Matsuya et al. were subsequently successful in showing that activation of the nuclear factor κB (NF-κB) p65 pathway signaled the occurrence of RIRE, while significant dual activation of the NF-κB p65 and cyclooxygenase 2 (COX-2) pathways signaled the occurrence of RIBE [55].

As noted by Yu [20], this was a very important step towards elucidating the origin of RIFSE. In fact, activation of different pathways for the occurrence of RIRE and RIBE might prove very useful in providing clear explanations to some previous intriguing results on RIFSE and RIRE. For example, when Ojima et al. found evidence of RIRE between irradiated and non-irradiated MRC-5 cells while studying RIFSE, they also noticed the fewer DSBs in the cells within the in-field area but in contact with the out-of-field area [17], which the authors attributed to the time spent by the bystander signals to diffuse from irradiated cells to non-irradiated bystander cells [75]. Moreover, Ojima et al. also recorded more Ki-67-positive cells just outside the in-field area, for all the studied radiation field size [17], where Ki-67 was a common marker for cell proliferation. Ojima et al. [17] suspected the migration of these Ki-67-positive cells from the out-of-field area, while Yu [20] further proposed that the extra Ki-67-positive cells were the result of proliferative bystander response which was a RIBE in the bystander cells. These hypotheses could be tested if the time-dependent activation of NF-κB p65 and COX-2 pathways in the cells could be ascertained through using the same microbeam irradiation setup as that employed by Ojima et al. [17].

### 4.5. FLASH Effect

Despite that proton therapy has been touted as one of the most advanced radiotherapy modalities, the proton FLASH effect has remained equivocal [80], which has been attributed to the limited range of accelerators that can deliver the required ultrahigh mean and bunch dose rates [22,81,82,83,84,85]. In relation, Patriarca et al. described the experimental setup and demonstrated the technical feasibility for FLASH-mode proton irradiation of small animals using a clinical cyclotron system [86].

Interestingly, the dose rates of microbeam protons generated by SPICE at NIRS delivered to studied cells and the corresponding dose delivery time appeared to fall into the FLASH effect regime as described above. As described in the Introduction and Section 3.2.4 above, the dose rates of microbeam protons delivered to some commonly used cells were in the order of 10^2^ to 10^3^ Gy s^−1^ [44], depending on the mass of the nucleus chosen in the determination of the absorbed dose. The desired proton doses (e.g., 500 protons per cell) could also be delivered in 0.05 s. As such, whether microbeam protons could also provide a platform to study the FLASH effect or whether the results on RIRE described in Section 3.2 above would represent the resultant effect from FLASH effect as well certainly deserve more research studies.

Moreover, as mentioned in Section 4.1 above, there has been research into MRT using microbeam radiations [76]. Interestingly, Fernandez-Palomo et al. further remarked that MRT could operate under the FLASH-mode with doses delivered within a few milliseconds and at ultrahigh dose rates (up to 16 kGy s^−1^), which could help spare normal tissues while efficiently eradicating local tumors [13]. As both the spatial fractionation provided by MRT and the ultrahigh dose rate of FLASH inflict lower toxicity to normal tissues, it is pertinent to examine the resultant sparing effect on normal tissues and the potentially improved tumor control of the FLASH-mode MRT when compared to conventional irradiation. In relation, it was established that normal tissues were better spared when the MRT dose rate was higher [25,28]. Apparently, research into FLASH-mode MRT would exploit many of the unique features of microbeam radiations, including the small beam size, facilitation of cell cultures with mixed targeted and non-targeted cells, well-defined irradiation areas, and ultra-high local doses and dose rates.

### 4.6. In Vivo Studies on RIRE—Zebrafish Embryos

In addition to in vitro studies, it is important that RIRE should also be confirmed in in vivo models that can provide predictions for humans, before RIRE can be considered for practical real-life applications such as radiotherapy including, e.g., development of alternative treatment procedures or drugs. As described in Section 3.2.4 above, microbeam protons provided by SPICE at NIRS were employed to irradiate dechorionated zebrafish embryos (*Danio rerio*) at the two-cell stage at 0.75 hpf, and RIRE between the irradiated cells and their non-irradiated counterparts in the embryos was verified [54]. To the best of our knowledge, this was the only in vivo model to date that was used in studying RIRE related to microbeam irradiation.

The popularity of zebrafish embryos as an in vivo model, particularly for studying non-targeted effects of ionizing radiation was reviewed by Yu and Cheng [87] and Choi and Yu [88]. In fact, zebrafish (*Danio rerio*) and its embryos have been established as popular vertebrate models in various research fields including, e.g., toxicology [89,90], developmental biology [91], and carcinogenesis [92]. Zebrafish enjoy a particular advantage as an in vivo model in that the zebrafish and human genomes share considerable homology, including conservation of most DNA repair-related genes [93,94]. In fact, about 70% of the human genes had at least one obvious zebrafish ortholog [94]. Moreover, zebrafish have other advantages as in vivo models since they have immune, hematopoietic, vascular, central nervous systems, and organs, which enable their potential use to study molecular mechanisms underlying human diseases [95]. The embryos are also practically convenient to use since they (1) are optically transparent to allow microscopic inspection; (2) develop rapidly to shorten experiments; (3) are produced in large numbers daily to facilitate high throughputs of experiments; and (4) can take up drugs directly from the medium.

In Section 4.5 above, the FLASH effect was described and discussed. Incidentally, as described in the Introduction and Section 3.2.4 above, the dose rates of microbeam protons at SPICE of NIRS delivered to some commonly used cells could reach 10^2^ to 10^3^ Gy s^−1^ [52], which fell into the FLASH effect regime. Although Choi et al. initially computed the dose absorbed by a single cell of the two-cell stage zebrafish embryo through the proton energy deposited into the entire cell [54], the local dose rate delivered to the cell would be commensurate with the nominal values of 10^2^ to 10^3^ Gy s^−1^ if the relevant mass was instead referred to that of the biological material traversed by the microbeam protons only (with microbeam diameter of 2 μm) [52]. As such, the results described in Section 3.2.4 above on in vivo studies on RIRE using zebrafish embryos could have represented the resultant effect from RIRE and the FLASH effect, and could have been an early serendipitous exploration of the in vivo FLASH effect. Apparently, studying RIRE or (RIRE + FLASH effect) using zebrafish embryos would exploit the small beam size, well-defined irradiation areas, and ultra-high local doses and dose rates provided by the microbeam radiations.

Beyreuther et al. reiterated the importance of confirming the FLASH effect in an in vivo model that possessed predictive values for humans, despite the success in demonstrating the FLASH effect in in vitro experiments [22]. To achieve this objective, Beyreuther et al. purposely set up a proton irradiation facility at the University Proton Therapy Dresden and established the corresponding beam parameters for irradiating zebrafish embryos with 224 MeV protons, either by a continuous beam with a conventional dose rate of 5 Gy.min^−1^ or by FLASH with a dose rate of 100 Gy s^−1^ (treatment time < 0.5 s). The zebrafish embryos were irradiated with graded proton doses at the pharyngula stage (~24 hpf), and the survival and morphological malformations were recorded daily for up to 4 d post-irradiation, after which the embryos were sacrificed and fixed for histological analyses. Intriguingly, Beyreuther et al. reported no significant differences in the survival and malformation rates of zebrafish embryos for different proton dose rates except that the rate of pericardial edema detected on the third and fourth day post-irradiation with a proton dose of 23 Gy delivered under the FLASH mode was significantly decreased when compared with the conventional mode [22]. The results were somewhat unexpected as the electron FLASH effect was confirmed in zebrafish embryos [96].

Beyreuther et al. [22] attributed the discrepancies to the different stages of the embryos used (24–28 hpf embryos for proton FLASH vs 4 hpf embryos for electron FLASH [96]) and the different energy deposition patterns of protons and electrons. Beyreuther et al. admitted that the 4 hpf zebrafish embryos might be more appropriate for verification of the proton FLASH effect but nonetheless noted the very tight time schedule from fertilization of the embryos to the irradiation. It might be worth noting that Choi et al. [54] identified ways to irradiate zebrafish embryos at 0.75 hpf (at the two-cell stage), after dechorionation, although the experimental setup at the two irradiation facilities might have major differences. It might also be worth noting that dechorionation of embryos could help determination of the energy and thus the ionization density of the protons when the latter reached the cells of the embryos.

## 5. Conclusions

(a) The current paper reviewed previous results on radiation-induced rescue effect (RIRE) between targeted irradiated cells and non-irradiated bystander cells obtained from microbeam experiments.

(b) RIRE was demonstrated in previous microbeam experiments, including: microbeam-proton-irradiated A549 cells rescued by bystander WI38 cells, microbeam-proton-irradiated NSCCs rescued by bystander CSCs, microbeam-proton-irradiated cells rescued by bystander cells in vivo on two-cell stage zebrafish embryos (*Danio rerio*), and microbeam-X-ray-irradiated MRC-5 cells rescued by non-irradiated MRC-5 cells.

(c) Microbeam irradiation systems at NIRS and KEK, both in Japan, were employed for previous studies on RIRE, and the unique features included the small beam size, well-defined irradiation areas, facilitation of mixing co-cultured targeted and non-targeted cells, and delivery of ultra-high local doses and dose rates. Nevertheless, the number of irradiated cells might be limited, and the high dose rate might make it challenging to delineate RIRE from FLASH effects.

(d) SPICE at NIRS delivered proton dose rates in the FLASH effect regime. Whether microbeam radiations can be exploited to study the FLASH effect or whether the results on RIRE obtained from microbeam experiments represent the resultant from (RIRE + FLASH effect) deserves more attention and research effort.

(e) Improved understanding on mechanisms underlying RIRE will enable better comprehension or prediction of radiotherapy treatment outcomes, as well as development of drugs or alternative treatment through controlling RIRE. One interesting proposal was to reduce the strength of RIRE through introducing extra irradiated cells to compete for the limited rescue signals.

(f) Dose responses for RIRE are important for deciphering the phenomenon, including the resultant from (RIRE + FLASH effect), and are crucial for better understanding or predicting treatment outcomes of chosen radiotherapy procedures.

(g) RIRE should be confirmed in in vivo models that can provide predictions for humans before RIRE can be considered for practical real-life applications.

## Figures and Tables

**Table 1:** Summary of studies by various research groups who demonstrated RIRE through different types of ionizing radiations, doses, combination of bystander/rescued cells, experimental end points and rescue responses.

Radiation Type	Doses to Irradiated Cells	Bystander/Rescued Cells	Rescue Responses/ Experimental End Points	Authors/Year/Ref
Broad-beam photons: 6 MV X-rays	2/4 Gy	NHDF/	micronucleus formation and extent of apoptosis reduced in irradiated cells	Widel et al., 2012 [43]
		Me45		
Broad-beam photons: ^137^Cs γ-rays	70/550 mGy	ZF4/ZF4	γ-H2AX foci reduced in irradiated cells	Pereira et al., 2014 [45]
Broad-beam photons: 200 kV X-rays (filtered)	2 Gy	HeLa/HeLa	autophagy accumulation reduced in irradiated cells	Kong et al., 2018 [34]
Broad-beam photons: 6 MV X-rays	1 Gy	(1) WI-38/ WI-38	γ-H2AX foci/nucleus reduced in irradiated cells	Matsuya et al., 2019 [46]
		(2) HBEC-3KT/ HBEC-3KT		
Broad-beam photons: 200 kV X-rays (filtered)	2 Gy	(1) HeLa/HeLa	mRNA expression levels of 53BP1 and PARP1, fluorescent intensity of PARP1 reduced in irradiated cells	Pathikonda et al., 2020 [47]
		(2) MCF7/MCF7		
		(3) CNE-2/CNE-2		
		(4) HCT116/ HCT116		
Broad-beam	1 mGy to 10 Gy	(1) WI-38/ WI-38	nuclear γ-H2AX foci in irradiated cells under non-uniform exposure decreased when compared to those in cells under uniform irradiation	Matsuya et al., 2019 [46]
(β-particles + γ-ray photons) from ^137^Cs- and ^134^Cs-bearing microparticle				
		(2) HBEC-3KT/HBEC-3KT		
Broad-beam	20/40 cGy	(1) NHLF/NHLF	53BP1 foci, micronucleus formation and extent of apoptosis reduced, while survival enhanced in irradiated cells	Chen et al., 2011 [14]
α-particles		(2) HeLa/HeLa		
Broad-beam	40 cGy	HL-7702/U937	micronucleus formation reduced in irradiated cells	He et al., 2014 [49]
α-particles				
Broad-beam	5 cGy	HeLa/HeLa	53BP1 foci reduced in irradiated cells	Lam et al., 2015 [50,51]
α-particles				
Microbeam-protons	~300 Gy	WI38/A549	γ-H2AX foci fluorescence intensity per nucleus reduced in irradiated cells	Desai et al., 2014 [44]
Microbeam-protons	~300 Gy	WI38/A549	γ-H2AX foci fluorescence per nucleus reduced in irradiated cells	Kobayashi et al., 2017 [52]
Microbeam-protons	~300 Gy	CSCs/NSCCs	relative fluorescence unit of nucleus 53BP1 foci reduced in irradiated cells	Liu et al., 2015 [53]
		of HT1080		
Microbeam-protons	~300 Gy	Cells/cells in vivo on two-cell stage zebrafish embryos (*Danio rerio*)	apoptotic signals similar to and different from background for 1-cell and two-cell irradiation of two-cell stage embryos, respectively	Choi et al., 2012 [54]
Microbeam-X-ray: synchrotron X-ray microbeam	1 Gy	MRC-5/MRC-5	53BP1 foci/cell decreased in small-size fields surrounded by non-irradiated cells	Ojima et al., 2021 [17]

**Table 2:** Data extracted from ref. [54]. Normalized values (normalized average numbers of apoptotic signals with respect to the average of the corresponding control samples) for 25 hpf zebrafish embryos that were exposed to different irradiation conditions at the two-cell stage (0.75 hpf). Irradiation conditions: first number represents the number of protons irradiated onto one cell of a two-cell stage zebrafish embryo; (×1) and (×2) represent one or both of the cells, respectively, were irradiated; (Exp 1) and (Exp 2) refer to two separate experiments. The numbers of apoptotic signals are compared with those detected on control samples, and the *p* values are obtained using *t*-tests.

Irradiation Conditions	Normalized Values	*p* Value (cf. Control Samples)
10 × 1 (Exp 1)	0.242	0.123
10 × 1 (Exp 2)	0.048	0.397
10 × 2 (Exp 1)	0.352	0.069 #
10 × 2 (Exp 2)	0.446	0.027 *
20 × 1	0.053	0.30
20 × 2	0.060	0.189
40 × 1	–0.028	0.450
40 × 2	0.631	0.034 *
50 × 1	0.429	0.095
50 × 2	0.192	0.210
80 × 1	0.152	0.479
80 × 2	0.363	0.050 *
100 × 1	–0.014	0.451
100 × 2	–0.220	0.066 #
160 × 1	0.335	0.017 *
200 × 1	–0.189	0.116
200 × 2	–0.298	0.022 *
300 × 1	–0.014	0.464
300 × 2	0.311	0.0968
2000 × 1	0.592	0.029 *
2000 × 2	0.283	0.0645 #

* cases with *p* ≤ 0.05, which are considered statistically significant; # cases with *p* > 0.05 and ≤ 0.07, which are considered marginally significant [54].

## Data Availability

Not applicable.
